# Reference Intervals for Serum Ferritin in Older Adults—Results from the Prospective SENIORLAB Study

**DOI:** 10.3390/jcm15083135

**Published:** 2026-04-20

**Authors:** Galina Ludin, Rita Maria Baron, Urs E. Nydegger, Marlene Jarquin Campos, Pedro Medina Escobar, Benjamin Sakem, Harald Renz, Karin Jung, Lorenz Risch, Martin Risch

**Affiliations:** 1 Dr. Risch Medical Laboratory, Sonic Suisse SA, Waldeggstrasse 37, 3097 Liebefeld, Switzerland; g.ludin@mailbox.org (G.L.); ursnydegger2@me.com (U.E.N.); pedro.medina@mcl-risch.ch (P.M.E.); benjamin.sakem@mcl-risch.ch (B.S.); karin.jung@risch.ch (K.J.); martin.risch@ksgr.ch (M.R.); 2 Center of Laboratory Medicine, University Institute of Clinical Chemistry, University of Bern, Inselspital, 3010 Bern, Switzerland; 3 Dr. Risch Medical Laboratory, Sonic Suisse SA, Wuhrstrasse 14, 9490 Vaduz, Liechtenstein; rch.baron@gmail.com; 4 Institute for Laboratory Medicine, Private University in the Principality of Liechtenstein, Dorfstrasse 24, 9495 Triesen, Liechtenstein; marlene.jarquin@ufl.li; 5 Institute of Laboratory Medicine and Pathobiochemistry, Molecular Diagnostics, Philipps University Marburg, Baldingerstrasse, 35043 Marburg, Germany; harald.renz@uk-gm.de; 6 Zentrallabor, Kantonsspital Graubünden, Loësstrasse 170, 7000 Chur, Switzerland

**Keywords:** reference intervals, reference limits, ferritin, older adults, seniors, geriatrics, indirect method, direct method

## Abstract

**Background**: Test descriptions from major diagnostic manufacturers do not include ferritin reference intervals (RIs) for individuals aged 60 and older. The absence of older adults-specific RIs contrasts with the widespread use of serum ferritin testing in older adults. We aimed to establish and verify RIs using two common analytical methods. **Methods**: For this study, 1467 older adults were prospectively enrolled and monitored for morbidity and mortality, and exclusion criteria were applied. Ferritin was measured using chemiluminescent microparticle immunoassay (CMIA) and transferred to an electrochemiluminescence immunoassay (ECLIA) using method comparison. RIs were evaluated using a direct method with a prospective observational study based on healthy individuals according to the Clinical and Laboratory Standards Institute (CLSI) 28-A3c guideline and compared with RIs obtained using an indirect approach based on data obtained in clinical routine outpatients, where normal and abnormal values are supposed to be statistically differentiated to determine RIs. When applied within a countrywide population-based setting in Liechtenstein, the impact of novel RIs on the frequency of abnormal values was analyzed. **Results**: A total of 386 men and 532 women were included in the direct RI determination. Women (W) had significantly lower ferritin levels than men (M), while age over the age of 60 years had no significant association with ferritin in men and women. RIs were 23–241 ng/mL (W) and 19–396 ng/mL (M) for CMIA and 27–293 ng/mL (W) and 23–480 ng/mL (M) for ECLIA. These RIs are higher than those mentioned in the test descriptions in both tests. In comparison, the indirect method for both assays showed comparably lower reference limits, whereas upper reference limits were only approximately similar. The prevalence of high abnormal ferritin levels was considerably lower with this study’s RIs compared with manufacturer RIs. **Conclusions**: Employing older adults-specific RIs in clinical routine seems to be advisable. This reduces the frequency of abnormal high values in comparison with the widely applied practice of extrapolating RIs obtained from younger age groups to older adults and therefore leads to fewer follow-up investigations.

## 1. Introduction

Iron is one of the most important trace elements in the human body. The body contains about 3–4 g of iron [1], of which the majority is present in hemoglobin and myoglobin for oxygen transportation throughout the body as well as for the oxygenation of muscle tissue. Another part of the body’s iron is storage iron, mainly ferritin, whose measurement in serum is also an effective and valid marker for evaluating iron stores [2,3].

A low ferritin (<15 ng/mL [2]) serum level is one of the first indicators of iron deficiency [4]. The World Health Organization (WHO) has recognized that iron deficiency is the most common nutritional deficiency globally and a major cause of anemia [4]. The DO-HEALTH trial [5] reported a prevalence of iron deficiency of 26.8% in older adults. Considering the high prevalence of iron deficiency, the accurate measurement and interpretation of ferritin serum levels in older adults are crucial for identifying the condition. However, there is a risk of false normal values in patients with iron deficiency, due to inflammation or other states of disease, which are discussed below [6,7,8].

An increased serum ferritin level can be considered an indicator of iron overload. Overload can be caused by hereditary or acquired factors, such as hemochromatosis or red blood cell transfusion, which can result in cardiomyopathies, joint disease, diabetes mellitus, and other secondary diseases [9]. Other reasons for increased ferritin concentrations can be metabolic syndrome [7,8], obesity [8,9,10,11], inflammation, liver disease [6], and malignancies [12]. However, high serum ferritin is insufficient for the identification of iron overload [12]; further laboratory and possibly invasive diagnostics are necessary to confirm the excess of iron. Whereas hemochromatosis at the time of diagnosis usually presents with high levels of serum ferritin (>1000 ng/mL), mildly elevated serum ferritin (<1000 ng/mL), especially with normal transferrin saturation, is more likely due to other causes [13,14] such as hepatic, renal, or neoplastic disease.

The interpretation of a ferritin level depends on comparing the result to a reference interval (RI). In routine laboratory practice, these RIs are often taken from the package inserts provided by assay manufacturers. This is performed despite the fact that it is recommended that every laboratory should verify and evaluate its own RIs.

According to a relevant guideline, the Clinical and Laboratory Standards Institute (CLSI) guideline EP 28-A3c [15], the preferred method for establishing RIs is the direct method. Direct methods use specifically designed prospective studies in a healthy study population. This approach is very resource-intensive and can be difficult to conduct in special populations (e.g., neonates, children, older adults) because of difficulties in recruiting a sufficient number of individuals or the high prevalence of disease and medications exerting an influence on laboratory values (e.g., in older adults) [16].

To establish RIs more conveniently, so-called indirect methods employing routine clinical laboratory data for computational processing of RIs have been developed and are becoming increasingly popular. Such indirect methods have also been adopted by the CLSI EP 28A3c guideline [17]. Indirect methods are somewhat sensitive to the prevalence of a specific disease, potentially introducing bias in the estimation of RIs [16]. One approach for validated RI determination in older adults (where the prevalence of comorbidity is high) has thus been to determine RIs by direct methods and to verify these RIs with indirect methods, which was recently completed for platelet counts and platelet indices in the SENIORLAB study [18].

The difficulties in determining RIs in different subgroups might, inter alia, be the reason why major diagnostic test manufacturers do not endorse RIs for older adults or children in their package inserts for ferritin (Table 1). In clinical laboratories, a commonly encountered practice is that the RIs for ferritin in adults aged 60 years or less are extrapolated to older adults, which can introduce significant bias and therefore lead to potentially unnecessary follow-up investigations and medical resource wastage due to falsely identified iron overload or deficiency. Within the framework of the SENIORLAB study, we set out to fill this gap and determine RIs for serum ferritin for older adults aged 60 years and older using a direct method for two different immunological assay formats. We further verified these RIs using indirect methods. Finally, we aimed to determine the frequency of abnormal ferritin concentrations among older adults in a population-based setting in the principality of Liechtenstein and changes in prevalence according to different RIs.

## 2. Methods

### 2.1. Recruitment of Study Participants

#### 2.1.1. Cohort for Direct Determination of RIs

**Figure 1:**
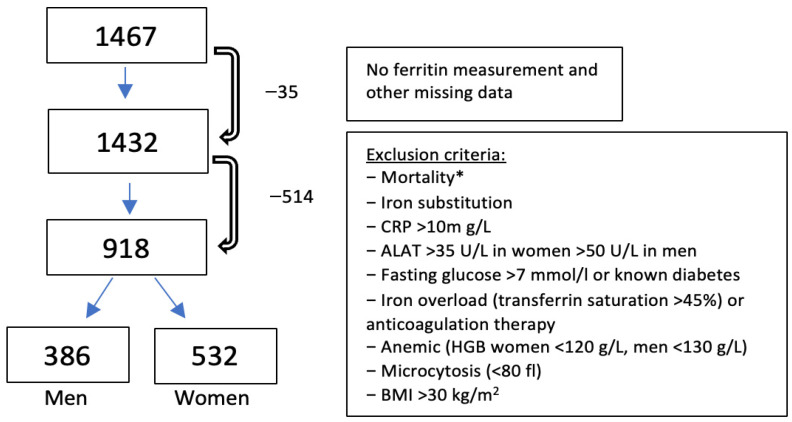
Exclusion criteria for serum ferritin RI estimation, which was performed in a final cohort of 386 men and 532 women. * Exclusion regarding time lag between ferritin measurement and death: >90 years: survival less than 1 year after measurement; 85–90 years: survival less than 2 years; 80–84 years: survival less than 3 years; <80 years: every death during follow-up.

Within the SENIORLAB study, 1467 Swiss residents aged 60 years and older (787 women and 679 men) were included, and multiple laboratory parameters were measured, as described in the study protocol published elsewhere [19]. Inclusion criteria were age 60 or older, subjective perception of being in good health, and an overnight fasting state at the time of baseline examination. The definition of health is challenging in older adults, since comorbidities become more common with age. The small percentage of older adults who do not have any comorbidities and are healthy as it is defined for younger adults is therefore not necessarily representative of this age group. The definition of “healthy” in older adults was therefore newly defined after excluding the following criteria: known diabetes, thyroid disease, current glucocorticoid use, active neoplastic disease in the past 5 years, polypharmacy (>5 pharmacologically active substances), and hospitalization during the past 4 weeks [19]. A follow-up was conducted 3 to 5 years later to elucidate health status, illness, or death. At older ages, survival can be regarded as an additional indicator of health. As already performed elsewhere [18], we therefore applied age-specific mortality criteria as exclusion criteria, as shown in Figure 1. Participants with no ferritin serum level measurement or patients taking iron substitution medication (oral and IV) were excluded. Further exclusion criteria were: indications of systemic inflammation (obesity with BMI >30 kg/m^2^ [20], CRP >10 mg/L [21]), factors known to influence serum ferritin levels (hepatic damage, diabetes, anemia, iron overload, anticoagulation therapy due to higher risk of bleeding, and microcytosis where iron deficiency is often present), see Figure 1 for an overview. All study participants provided written informed consent. The study was conducted in accordance with the Declaration of Helsinki and was approved by the cantonal ethics committee (Kantonale Ethikkommission Bern; ref 166/08; dated 27 October 2008). In the International Standard Randomized Controlled Trial Number registry, this study has been registered with the protocol identifier ISRCTN53778569.

#### 2.1.2. Cohorts for Indirect Determination of RIs

Data collection for the indirect method was performed within historical clinical routine data from the Dr. Risch group of medical laboratories. Ferritin measurements collected from 1 October 2004 to 31 January 2024 were included in the study. The results were anonymized such that only the following variables were available for data analysis: gender, month and year of blood draw, age at the time of blood draw (in full years), hospitalization status (yes/no), and the specialty of the treating physician. Duplicates were excluded by retaining the first value [22] measured for each patient for further indirect RI determination. Because hospitalized patients have a higher prevalence of diseases affecting serum ferritin concentrations and because indirect RI determination is sensitive to the prevalence of diseased individuals within a population [16], hospitalized inpatients and hospital outpatients were excluded from indirect RI calculation. Thus, only patients from outpatient healthcare providers were included in indirect RI determination. To verify the RIs obtained with direct methods, we used the age and gender partition employed in the direct RI determination within the SENIORLAB cohort. The ethics committee of the Canton of Bern waived the need for informed consent and approved the indirect determination of RIs in routine results (KEK Bern; BASEC 2020-00139; dated 17 March 2020).

### 2.2. Determination of Frequency of Abnormal Ferritin

With the establishment of age-specific RIs for the older adults, it is possible to determine the frequency of abnormal values in the population. During the period 1st January 2013 until 31st December 2021, a total of 15,384 (9467 women and 5914 men, corrected for death cases) older adults aged 60 years or more lived in the Principality of Liechtenstein. Serum ferritin data in the laboratory database in the period from 1 January 2013 to 31 December 2021 revealed a total of 34,232 ferritin determinations. After the removal of duplicates, with the first values of a respective patient with multiple determinations remaining, there were 8215 patients with available serum ferritin values (4791 women, 50% of the country’s age-specific total female population; 3426 men, 58% of the age-specific total male population). The protocol for this part of the study was verified by the ethics committee of the Kanton of Zurich (KEK Zürich, Zürich, Switzerland; BASEC 2020-00918; dated 30 July 2020), and informed consent was waived.

### 2.3. Laboratory Methods

There were two laboratory assay methods used in this study. The measurements of the SENIORLAB study, as well as the measurements utilized for the indirect method of RI calculation until 2012, were collected using chemiluminescent microparticle immunoassay (CMIA, ABBOTT Architect i2000; Abbott Diagnostics, Baar, Switzerland), which is calibrated to the first ferritin international standard 80/602. Measurements for the indirect approach from 2012 onwards, as well as the population group of the principality of Liechtenstein, were measured using electrochemiluminescence immunoassay (ECLIA, Roche COBAS 6000, Roche Diagnostics GmbH, Mannheim, Germany), which was also calibrated to the first ferritin international standard 80/602. According to our measurements, the coefficients of variation were 5.8% (at a concentration of 27 ng/mL) and 4.7% (at a concentration of 197 ng/mL) for the CMIA, and 3.9% (at a concentration of 32 ng/mL) and 4.4% (at a concentration of 202 ng/mL) for the ECLIA. The laboratory consistently participated in an external quality assessment program (Centre Suisse de Contrôle de Qualité (CSCQ), Chêne-Bourg, Switzerland) and maintained rigorous internal quality control using commercial control materials.

### 2.4. Statistical Methods

Continuous variables were summarized as the median and interquartile range (IQR). Normality was assessed using the Pearson D’Agostino test. Proportions were given as percentages together with their 95% confidence intervals. Correlations were assessed using Spearman’s rank correlations, and a comparison of medians was completed using the Kruskal–Wallis test.

For the determination of direct RIs, CLSI C28-A3c was applied [15]. Because ferritin does not follow a normal distribution, data was transformed using the Box–Cox method, turning the transformed results into a normal distribution. Tukey’s method was used for outlier detection and elimination, while 95% double-sided RIs with their 90% confidence intervals were established using the “robust method” (CLSI C28-A3c [15]). Age-related continuous RIs were calculated based on the Altman method [23].

**Figure 2:**
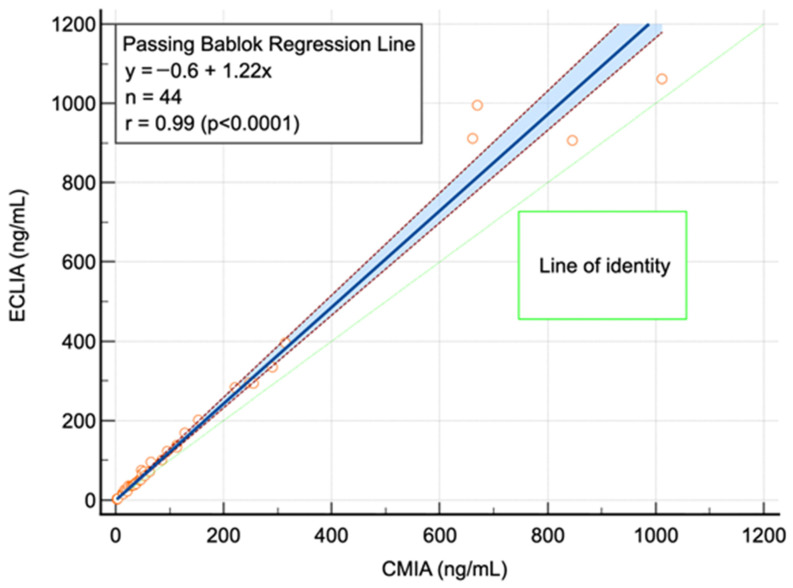
Method comparison for serum ferritin (ng/mL) with ECLIA and CMIA employing Passing–Bablok regression. r—correlation of coefficient, n—number of investigated individuals.

The Harris and Boyd method was employed to elaborate differences in RIs in gender- and age-stratified groups. Hence, such subgroup analysis was made in a spreadsheet using Box–Cox transformed data (CLSI C28-A3c [15]). Medcalc^®^ (Medcalc software, Brussels, Belgium, Version 22.017, January 2024) and Microsoft Excel (MS Excel, Microsoft, Seattle, WA, USA, Version 2312, January 2024) were used for statistical analysis. To allow for the transference of direct RIs from one method (CMIA) to another (ECLIA), we conducted a method comparison. Passing–Bablok regression analysis was performed for this purpose (Figure 2), and the following regression equation was obtained to transfer the measurements (ECLIA = −0.6 + 1.22 × CMIA). Upon visual inspection, the relationship between the two methods appears to be linear within the concentration range shown in Figure 2.

To validate the RIs obtained with the direct method, an indirect estimation of RIs was performed with refineR [24], which is freely accessible on the statistical program package “R” (v4.3.2.; R Core Team, October 2023). R: A language and environment for statistical computing. R Foundation for Statistical Computing, Vienna, Austria. URL https://www.R-project.org/, accessed on 4 March 2024). In brief, refineR is a reference limit estimator using real-world data, which is composed of pathological and non-pathological samples as input. RefineR identifies the hidden Gaussian distribution representing healthy individuals through transformation, likelihood optimization, and tail down-weighting, and then derives double sided 95% RIs and 95% confidence intervals with the bootstrapping method from this estimated distribution.

### 2.5. Literature Search for Ferritin RIs

To compare the obtained RIs with other works, we performed a literature review of Pubmed until 31 July 2025. The search was performed with the following search terms: ferritin OR iron OR iron status AND normal range OR reference range OR reference interval OR normal interval AND older adults OR age OR senior AND healthy. The cited literature from the selected publications was searched additionally. Literature older than 1985 was excluded since the first international standard for serum ferritin was released in 1985. In this systematic review, we only included reports using methods calibrated to the first international standard 80/602 and ECLIA/CMIA assays.

## 3. Results

### 3.1. Determination of RIs Using the Direct Method

After applying exclusion criteria, a total of 918 individuals (532 women, 386 men; median age 71 years) were included in the present study. Baseline characteristics from the study participants can be seen in Table 2.

Men had significantly higher ferritin concentrations than women (*p* < 0.0001). Sex and age were partitioned (according to decades) before determining RIs. As can be seen in Table 3, the lower reference limits (LRLs) and upper reference limits (URLs) were similar between the three age strata, both in women and men. There were no significant differences in serum ferritin concentrations between the three age strata (all Z < Z*, according to Harris and Boyd, no partitioning), both in men and women. In addition, the correlation between ferritin and age was not significant in both men and women.

**Figure 3:**
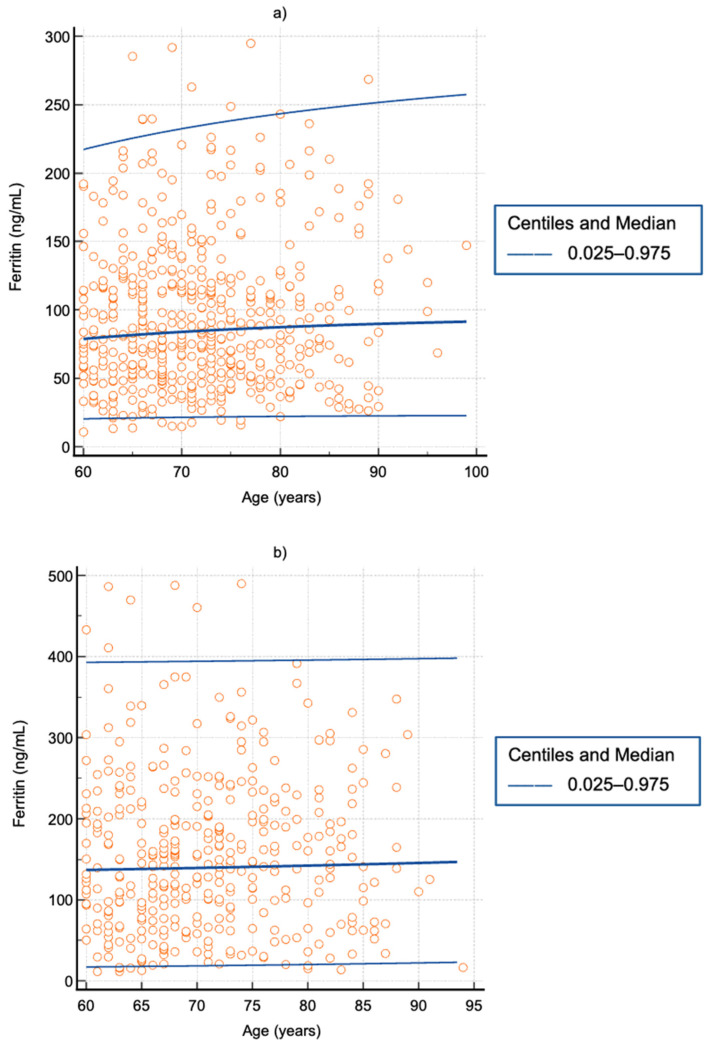
Continuous age-related reference intervals for serum ferritin in older adults aged 60 years or more, as determined using CMIA: (**a**) women; (**b**) men.

Furthermore, when applying the Harris and Boyd method, the RIs among the three age strata were not different for men or women. This is finally corroborated when looking at the continuous age-related RIs obtained using the Altman method (Figure 3).

Due to the fact that the three age strata did not differ and since the medians and the age-related continuous RIs stayed constant over age, a pooled RI determination among all women and all men was completed. With this, the serum ferritin RIs determined using the direct method from the results obtained from CMIA are shown in Table 4.

The ferritin measurements obtained with CMIA were transferred to ECLIA using the regression equation obtained from the method comparison, and RIs were established. Age- and sex-stratified serum ferritin values from ECLIA are shown in Table 5.

Analogous to RI determination for serum ferritin measured with CMIA, the transference of the common sex-stratified CMIA reference limits for women and men aged 60 years and more to ECLIA is shown in Table 6.

### 3.2. Determination of RIs Using the Indirect Method

RIs obtained from routine outpatients are provided in Table 7 (CMIA) and Table 8 (ECLIA). After first including all patients, we observed clinically implausible URLs in both CMIA and ECLIA, especially in men. With the hypothesis of a high prevalence of diseases leading to high serum ferritin concentrations in outpatients and the knowledge of inflammation being a frequent cause of increased ferritin concentrations, we then selected individuals with concurrent determinations of CRP values. When we only included individuals with CRP levels < 10 mg/L—which would be in line with our exclusion criteria for the study group of the direct determination of RIs—the URL in both methods was substantially lower. The URL was progressively lower when only individuals with CRP < 3 mg/L or even <1 mg/L were included. This indicates that a routine outpatient cohort of patients have a disease prevalence that might be too high to reliably estimate the URL for ferritin from routine data.

When comparing the reference limits of RI determinations with the direct method to those obtained with indirect methods performed for all patients, it can be seen that indirect RI determination shows an overlap at the lower CI, and thus, can verify the LRLs obtained with direct methods for both sexes with CMIA and for women in ECLIA. The 95% CI of men at the LRL of ECLIA obtained using indirect RL determination in all individuals (30 ng/mL) is just adjacent to the 90% CI of the LRL obtained using direct RL determination (i.e., 29 ng/mL).

When looking at the URLs obtained for CMIA, the 90/95% CIs in women overlap between the direct and the indirect methods when only women with CRP < 10 mg/L are included in the RI determination with the indirect method. When comparing the respective 90/95% CIs of the URL in men, no overlap can be seen. This does change when only men with a CRP < 1 mg/L are included (upper limit of 90% CI in URL with direct method 418 ng/mL vs. lower limit of 95% CI in URL with indirect method 321 ng/mL), although the sample size was crucially smaller than in the groups with higher CRP levels (N = 742) and the 95% CI was very wide (321–570 ng/mL). This raises the question of whether the number of samples was too low to indirectly estimate an accurate URL in men.

When comparing the 90/95% CIs of the URLs of direct and indirect methods obtained for ECLIA, the confidence intervals in women overlap only when including women with CRP < 1 mg/mL into RI determination with the indirect method. No overlap of CIs in URLs was observed at higher CRP concentrations or in the general population. In men, the upper limit of the confidence interval of the URL of ECLIA (i.e., 507 ng/mL) was somewhat lower than the respective lower limit of the confidence interval of the URL from RI determination with the indirect method (i.e., 532 ng/mL) in men with a CRP < 1 mg/L, hence it did not exhibit an overlap. However, the difference in the two confidence intervals limits only show ≈5% difference. In conclusion, indirect RI determination can confirm the LRL of direct ferritin RI determination in both men and women with CMIA and ECLIA. However, at the URL, such a confirmation can only be partly obtained when selecting patients with low CRP levels and allowing a 5% difference in the adjacent limits of the CI.

### 3.3. Systematic Review of RIs Reported in the Literature

Sources investigating RIs with analytical ECLIA/CMIA methods traceable to the first international standard 80/602 were included. There was only one study using the same international standard, assay method, and direct calculation of RIs in older adults aged 60 years and older [25]. The RI-specific data of that study by Wang et al. is shown in Table 9.

### 3.4. Impact of Introducing Older Adults-Specific RIs

**Figure 4:**
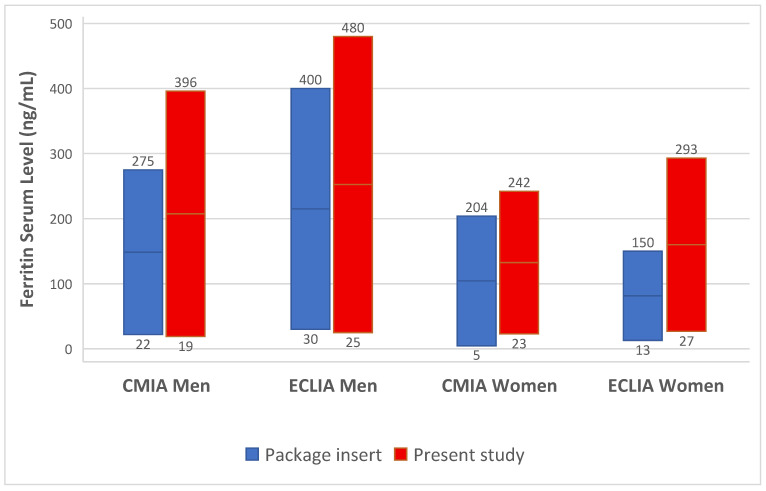
Reference intervals with their URLs and LRLs suggested in package inserts for CMIA and ECLIA are shown in blue. The RIs obtained for older adults in this study using the direct determination method are shown in red.

A summary of the RIs or expected values for normal adult persons from the package inserts of the investigated assays, together with the RIs obtained for older adults from the direct RI determination, is shown in Figure 4.

Different RIs are expected to result in different prevalences of abnormal results. Because of this, we applied the RIs from the direct determination of ECLIA to the ferritin results obtained in older adults ≥60 years in the principality of Liechtenstein to allow for the reference limit-specific quantification of abnormal values in a population-based approach. Ferritin concentrations have been measured using the same method by one laboratory. During the observation period, 60% of the entire population aged 60 and older had at least one ferritin measurement collected. The prevalence of abnormal values was analyzed using the RIs obtained in this study and compared to the RIs given by the package inserts for adult individuals aged 18–60 years (in the absence of any suggestion for older adults-specific RIs). The respective RLs from the package insert are 30–400 ng/mL for men and 13–150 ng/mL for women.

The prevalences of low and high ferritin, interpreted according to conventional package insert RLs, are shown in Table 10. The total frequency of abnormal ferritin values is 39% in women and 25% in men. The abnormal cases are mainly due to high ferritin values, with the prevalence of low ferritin values being in low, single-digit percentages.

The prevalence of abnormal ferritin concentrations when using RIs obtained for ECLIA in the present study is shown in Table 11. It can be seen that abnormal ferritin concentrations in women are present in 16% of the country’s ferritin determinations, whereas the respective prevalence in men was 18%. The frequencies of abnormal ferritin concentrations are mainly due to increased ferritin. Together, applying age-specific RIs for ferritin in older adults resulted in a relative reduction in abnormal ferritin concentrations by nearly 60% (16% vs. 39%) in women, whereas the same relative reduction in men was nearly 30% (18% vs. 25%).

## 4. Discussion

Major assay manufacturers provide limited information on RIs for individuals aged 60 and older. In this study, we established RIs for serum ferritin in the older adult population, where ferritin is among the most frequently ordered tests. We evaluated two widely used assay methods—CMIA and ECLIA—both calibrated to the same international standard (80/602). Despite this shared calibration, the results differed between methods, highlighting the need for method-specific RIs. Moreover, the RIs established in this study differed substantially from those reported in package inserts. From a methodological perspective, we also found that indirect RI determinations may be unreliable for serum ferritin, particularly at the URLs. This suggests that ferritin is among the parameters for which indirect RI determination may be inappropriate.

When examining the expected values or RIs provided in the package inserts of ferritin assays, significant heterogeneity becomes apparent. None of this information covers the full age range of potential patients (i.e., newborn to centenarians), some fail to specify the population from which the RIs were derived, and others use insufficient sample sizes (i.e., fewer than 120 individuals per stratum) for direct RI determination. Regarding methodology, there is also considerable variation: some inserts report a 90% RI, others a 95% RI, while some use non-CLSI methods to define a range (e.g., geometric mean ± 95% confidence interval). Additionally, package inserts indicate that the reported RIs or expected values were established using a different analytical device or technology than the assay actually described in the insert itself. Although all package inserts state that each laboratory should establish its own RIs, it is common practice for laboratories to adopt the provided values without critically evaluating their validity [26]. In the case of ferritin, another frequent practice is extrapolating RIs to age groups not originally included in their determination, such as applying adult RIs to younger children or extending adult RIs to older adults. This study addresses these gaps by including a sufficiently large and well-defined cohort of reference individuals and employing a standardized, CLSI-recommended method for RI determination.

There are currently four WHO international standards for ferritin determination. The first WHO International Standard, 80/602, was released in 1985 and contained ferritin obtained postmortem from human liver [27]. The second WHO International Standard, 80/578, introduced in 1994, used human spleen ferritin from splenectomized patients who suffered from transfusion-related iron overload [28]. The third International Standard, 94/572, a recombinant ferritin L-chain preparation, was released in 1997 [29]. Most recently, in 2021, the fourth WHO International Standard, 19/118, a recombinant ferritin preparation traceable to the third International Standard, has become available [30]. Recent reports on ferritin measurement harmonization indicate that standardization is far from optimal [31,32,33] and that the disagreement of RIs due to a lack of harmonization is a major concern for clinical decision making [34,35]. Our study further highlights this issue, demonstrating differences between two chemiluminescence methods, despite both being calibrated to the same international standard. This underscores the need for method-specific rather than solely calibrator-based RIs, at least until comprehensive international harmonization with recalibration is achieved. Achievements in the harmonization of serum ferritin could maybe be improved by introducing practice guidelines [36] or through big data analytics [37,38], as done with other analytes.

Regarding the LRLs, we have found similar concentrations contained in package inserts. The only comparable study regarding a target population of older adults, study method, assay method, and the use of the first international standard has been reported by Wang et al. [25]. We found similar LRLs in men (23 vs. 24.4 ng/mL), whereas we found substantially lower LRLs in women (27 vs. 48.2 ng/mL) [25]. These differences, however, could be explained inter alia by the fact that the study by Wang et al. was performed with a Han population living in the Shanghai region, China, whereas our study was performed with individuals of Caucasian origin living in central Europe.

Jäger et al. [39], based on a retrospective analysis of a large primary care cohort, advocated for harmonization in diagnostic criteria for iron deficiency, which may be different from decision limits obtained from RL determination. However, Truong et al. [40] observed, in a systematic review regarding RIs for healthy adults, that the use of an LRL that was too low regarding serum ferritin was the result of the inaccurate identification of people at risk of iron deficiency in the study population, as well as the lack of adherence to RIs according to the CLSI. Our LRL is comparable with another independent study in men, whereas we cannot replicate the finding that women have a higher LRL than men [25]. The present study suggests a similar LRL for older adults, independent of sex. The LRL identified in this study is comparable, although somewhat lower (24 ng/mL), to those provided by manufacturers’ reagent package inserts for men aged < 60 years (30 ng/mL). However, our LRL for women (27 ng/mL) is substantially higher than in package inserts (13 ng/mL) for premenopausal women aged 60 years or less. Applying the LRL from this study instead of those of the manufacturer would thus lead to an increase in frequencies of diagnosed iron deficiencies in female older adults and to a lower frequency in male older adults. This is in agreement with Martens et al. [41], who postulated that commonly used LRLs lead to the underdiagnosis of iron deficiency, especially in women. The reason for an increase in female older adults’ LRL to levels known from male older adults can probably be attributed to the absence of recurrent blood loss after menopause [41].

At the URL, we noticed larger differences obtained in the present study when compared with values contained in package inserts and the published literature. While we saw a higher URL than the package inserts providing information for individuals 60 years and younger, Wang et al. reported an even higher URL than that found in our study. One reason for this might be ethnic differences in serum ferritin level [42], which indicates that different ethnic populations (such as Europeans, African Americans, South- and East Asians) cannot use each other’s RIs for serum ferritin. Further genetic basis of differences regarding iron metabolism would be needed to explain such differences.

Furthermore, age can also be an associated factor: higher serum ferritin levels are associated with hepatic, renal, hematologic, malignant, and metabolic disorders [14], which are all more common in older adults even though we only included healthy older adults, the frequency of such subclinical diseases is higher than in the younger population and might affect the URL for older adults during the determination of RIs. Iron overload, on the other hand, is relatively uncommon, especially in the presence of normal transferrin saturation [14]. The present study set iron overload, indicated by a transferrin saturation of >45%, as an exclusion criterion. When iron overload is ruled out in the clinical workup of patients, hyperferritinemia should be further investigated regarding infectious, hepatic, renal, or malignant diseases [43].

We noticed higher URLs for the older adults than for younger populations (when derived from package inserts), as reported previously in several studies [25,44,45,46]. Serum ferritin is known to be higher, especially for postmenopausal women, because menstrual blood loss has ceased [25,45]. The higher URL found in this study, when compared with the URL employed in younger individuals, suggests that introducing such older adults-specific RIs, as obtained in this study, in clinical routine would result in lower resource usage than if conventional RIs obtained from younger reference individuals are employed. In brief, we calculated RIs that can be applied to the older adult population in Switzerland and most likely in countries with a similar ethnic background, leading to a smaller percentage of older adult patients with abnormally high ferritin.

Besides the physiological determinants of RIs, such as age and sex, a further aspect, namely the method of RI determination, has an impact on the URL and LRL. In daily laboratory practice, the indirect method to establish RIs has become increasingly popular. This is due to the fact that data collection is much easier, and statistical tools to derive RI from routine data are increasingly available. Last but not least, studies to evaluate RIs using the direct method are much more costly than those using indirect methods. In this study, we compared direct and indirect calculation methods for serum ferritin within identical population groups. We observed a good agreement on LRL, whereas the URLs were much higher when calculated with the indirect method. We were able to show that, with the exclusion of participants with an increasing activity of inflammation markers, as seen with CRP, the URLs of ferritin progressively became lower. However, even with the exclusion of inaccurately increased ferritin concentrations due to inflammation, the URLs did not agree with the URLs calculated using the direct method. This indicates that other disorders affecting ferritin levels, such as subclinical liver cell damage in outpatients, probably exert an influence on the URL, because these disorders display a considerable frequency of presumably subclinical disturbed physiology in older adults. This raises the question of the accuracy of the indirect method in older adults, especially with analytes that are affected by other conditions, such as inflammation or hepatic or renal disease (which have high frequency in older adults), in the case of serum ferritin. A high proportion of sick people has been described as being associated with a lower accuracy of RI determination using indirect methods [17,47], therefore indicating the need to further investigate data cleaning before applying the indirect method [48]. The SENIORLAB study offers a framework to see which parameters provide an overlap of RIs obtained using direct and indirect methods, and thus, make indirect methods more suitable for the determination of solid RIs in older adults. Together, indirect methods do not seem to be reliable for determining ferritin RIs in older adults.

In this study, we were able to show that the prevalence of pathologic ferritin values can be substantially lowered in a population-based setting in the principality of Liechtenstein when applying the RIs obtained in the current study instead of manufacturers’ cutoffs extrapolated to older adults. With this, the abnormal values above the URL could be lowered in women by 71% and in men by almost 30%. The trend of the rising URL of ferritin in older adults has been previously observed [25]. This reduced percentage of abnormal values leads to a substantially lower rate of follow-up investigations and therefore to less medical resource wastage in older adults.

The present study has its limitations. First, there was no information about the dietary lifestyle of study participants available, such as a vegetarian or vegan diet. Second, the RIs for ECLIA were not calculated with directly measured values but obtained from the transference of the values from one method to the other (CMIA to ECLIA). Nevertheless, this method is recommended by the CLSI EP28 3Ac guideline when evaluating RIs for an alternative method. Third, the reference intervals (RIs) reported here are valid only for the analytical methods investigated. It is important that these RIs are not applied to methods other than those used in the present analysis. To avoid interpretative confusion, we therefore recommend that laboratory reports state not only the parameter name “ferritin” but also the analytical method used to determine the result (e.g., Ferritin (Roche ECLIA) or Ferritin (Abbott CMIA)). Fourth, although we postulate that ferritin RLs cannot be accurately evaluated in older adults, we cannot prove that the direct method is absolutely correct. As the definition of health in older adults is difficult, we not only conduct a cross-sectional characterization of individuals but also introduce survival as a potentially corroborative indicator of former health when blood was taken. This represents an additional feature of the SENIORLAB study and, in our eyes, strengthens the validity of the evaluated RLs. In sum, we believe that these limitations do not invalidate our findings.

## 5. Conclusions

There are several calibrators available for different methods; however, not even methods calibrated to the same international standard provide comparable results. For the time being, it is thus mandatory to provide method- and not standard-specific determinations of RIs. Furthermore, it is suggested that age-adapted RIs for serum ferritin measurements should be introduced in laboratory medicine practices for older adults. This will lead to considerably fewer follow-up investigations and therefore helps to minimize resource wastage for older adults. Finally, it seems advisable to initiate more studies comparing direct and indirect methods for establishing RIs in older adults to prevent the introduction of potentially erroneous RI limits in this patient group, giving rise to potentially incorrect medical decisions.

## Figures and Tables

**Table 1:** Overview of reference intervals/expected values of different assay methods for the determination of serum ferritin, as provided in the package inserts of four major test formats. CMIA—chemiluminescent microparticle immunoassay; ECLIA—electrochemiluminescence immunoassay, CLIA—chemiluminescence immunoassay. Data sources: CMIA: Architect Ferritin, Revised May 2010; ECLIA: Roche Elecsys: 2023-12, V 7.0 English; CLIA: Siemens Atellica IM Ferritin (Fer), Rev. 03, 2019-07; Paramagnetic CLIA: Beckman–Coulter: Instruction for use C64647 D, August 2024; RI—reference interval, CI—confidence interval, y—years, LRL—lower reference limit, URL—upper reference limit.

Assay Method	Laboratory Method for RI Evaluation	Reference Sample Group	Reference Interval and CI	Age Stratification	Male							Female						
					Age Range (y)	LRL	URL	Median	Samples N	Lower CI	Upper CI	Age Range (y)	LRL	URL	Median	Samples N	Lower CI	Upper CI
CMIA, ABBOTT Architect	AxSYM ferritin assay	“Normal”	Central 95% interval	No	N/A	21.8	275	75.6	32	N/A	N/A	N/A	4.6	204	39.4	60	N/A	N/A
ECLIA, Roche COBAS	Enzymun test	“Healthy”	5th and 95th percentiles	Yes	20–60	30	400	N/A	120	N/A	N/A	17–60	13	150	N/A	104	N/A	N/A
CLIA, Atellica, Siemens	ACS:180 system	Apparently healthy subjects with normal liver function enzyme tests, bilirubin, and serum iron tests	95th percentile range	No	N/A	22	322	94	142	N/A	N/A	N/A	10	291	46	134	N/A	N/A
Paramagnetic particle CLIA, Beckman–Coulter	Access ferritin	Apparently healthy male and female subjects	Not specified (specified as geometric mean plus minus 95% non-parametric CI)	No	N/A	23.9	336.2	105.6	49	N/A	N/A	N/A	11	306.8	51.4	64	N/A	N/A

**Table 2:** Baseline characteristics for study participants of the SENIORLAB study included in the reference interval determination using the direct method. Median is shown together with interquartile ranges (IQRs) shown in brackets.

Characteristics	Women (N = 532)	Men (N = 386)
Age (years)	71 (66; 77)	70 (65; 76)
Body mass index (kg/m^2^)	23.9 (21.9; 26.1)	24.9 (23.3; 26.9)
Systolic blood pressure (mmHg)	146 (130; 162)	147 (133; 162)
Diastolic blood pressure (mmHg)	90 (81; 100)	90 (80; 98)
Ferritin (ng/mL)	85 (53;123)	150 (85; 221)
C-reactive protein (mg/L)	1.3 (0.7; 2.4)	1.2 (0.6; 2.2)
Iron (µMol/L)	18.7 (16.0; 21.6)	19.1 (16.2; 23.0)
Transferrin (g/L)	2.6 (2.4; 2.8)	2.6 (2.4; 2.8)
Transferrin saturation (%)	28.7 (23.8; 33.8)	30.0 (25.2; 36.2)
Soluble transferrin receptor (mg/L)	1.11 (0.98; 1.26)	1.15 (1.0; 1.3)
Hemoglobin (g/L)	137 (131; 142)	149 (143; 155)
Fasting glucose (mmol/L)	5.1 (4.8; 5.4)	5.4 (5.0; 5.7)
ALAT (U/L)	16.0 (14; 20)	20.0 (16; 26)
ASAT (U/L)	21.0 (19; 24)	22.0 (19; 26)
Gamma glutamyltransferase (U/L)	17.0 (13; 23)	25.0 (19; 34)
Alkaline phosphatase (U/L)	67.0 (57; 80)	64.0 (56; 75)
Platelet counts (10^3^/µL)	243 (212; 278)	212 (185; 239)
Lactate dehydrogenase (U/L)	350 (323; 382)	328 (297; 360)
Albumin (g/L)	43 (42; 45)	43.2 (42; 45)
Low-density lipoprotein (mmol/L)	4.0 (3.4; 4.7)	3.6 (3.0; 4.2)
High-density lipoprotein (mmol/L)	1.85 (1.6; 2.2)	1.5 (1.3; 1.8)
Triglycerides (mmol/L)	1.1 (0.9; 1.5)	1.2 (0.9; 1.5)

**Table 3:** Sex- and age-stratified RIs for serum ferritin (ng/mL) in older adults obtained with CMIA. N—number of reference values. The 90% confidence intervals (CIs) are displayed in brackets.

CMIA	Women		Centiles of Ferritin		Men		Centiles of Ferritin	
Age (y)	N	Median	0.025	0.975	N	Median	0.025	0.975
60–69	225	83	21 (18–25)	238 (216–259)	179	132	18 (13–24)	407 (370–447)
70–79	199	85	25 (22–29)	220 (201–239)	134	161	25 (17–37)	390 (358–423)
≥80	94	86	22 (18–28)	263 (230–299)	63	141	11 (4–25)	389 (342–437)

**Table 4:** RIs for serum ferritin (ng/mL) for older adults aged 60 years and more, as obtained with CMIA.

CMIA	N	Median	LRL 90% (CI)	URL 90% (CI)
MEN	376	146	19 (15–25)	396 (374–418)
WOMEN	522	84	23 (21–25)	241 (228–255)

**Table 5:** Sex- and age-stratified RIs for serum ferritin (ng/mL) in older adults obtained using ECLIA.

ECLIA	Women		Centiles of Ferritin (CI 90%)		Men		Centiles of Ferritin (CI 90%)	
Age (y)	N	Median	0.025	0.975	N	Median	0.025	0.975
60–69	225	101	25 (21–29)	289 (265–314)	179	160	21 (15–29)	494 (449–542)
70–79	199	103	30 (26–34)	266 (244–290)	134	195	30 (20–45)	474 (435–514)
≥80	94	104	27 (22–33)	319 (279–363)	63	171	13 (5–29)	473 (415–530)

**Table 6:** RIs for serum ferritin (ng/mL) using ECLIA in older adults aged 60 years and older.

ECLIA	N	Median	LRL 90% (CI)	URL 90% (CI)
MEN	376	177	23 (18–29)	480 (454–507)
WOMEN	522	102	27 (21–30)	293 (276–310)

**Table 7:** CMIA indirect method for RIs for older adults >60 years. RL calculated with 200 bootstraps.

CMIA	Women	Centiles of Ferritin (CI 95%)		Men	Centiles of Ferritin (CI 95%)	
	N	0.025	0.975	N	0.025	0.975
All	17,719	16 (12–24)	353 (307–414)	9955	21 (7–35)	691 (546–827)
CRP < 10	3428	14 (10–20)	249 (212–312)	2280	22 (16–32)	547 (461–611)
CRP < 3	2465	13 (10–17)	222 (197–268)	1699	19 (13–44)	528 (476–588)
CRP < 1	1050	8 (5–18)	193 (163–264)	742	8 (7–33)	494 (321–570)

**Table 8:** ECLIA indirect method RIs for older adults >60 years. RL calculated with 200 bootstraps.

ECLIA	Women	Centiles of Ferritin (CI 95%)		Men	Centiles of Ferritin (CI 95%)	
	N	0.025	0.975	N	0.025	0.975
All	125,373	30 (23–31)	509 (427–511)	80,317	34 (30–48)	866 (824–936)
CRP < 10	15,101	21 (20–29)	416 (393–497)	9309	28 (22–47)	818 (633–907)
CRP < 3	10,862	26 (21–27)	441 (343–452)	6941	30 (22–44)	803 (594–857)
CRP < 1	4978	22 (18–27)	383 (297–432)	3193	30 (16–35)	740 (532–739)

**Table 9:** Results of systematic literature review: Wang et al.—1st International Standard (80/602), assay method Cobas 8000 e602 (Roche Diagnostics), direct determination of RIs.

Sex, Age Range (y)	N	LRL 95% (CI)	URL 95% (CI)	Median
Men > 60	173	24.4 (12.7–41.2)	627.1 (509–697.7)	215.1
Women > 60	256	48.2 (45.0–63.2)	554.9 (476.6–661.7)	176.7

**Table 10:** Frequency of abnormal ferritin values in a population-based setting of older adults from the principality of Liechtenstein with conventional RIs employed from the package insert from the ECLIA assay (women: 13–150 ng/mL, men: 30–400 ng/mL).

Sex	Age (y)	N	Abnormal N	Abnormal %	Low Ferritin N	Low Ferritin %	High Ferritin N	High Ferritin %
Female	60–69	2557	949	37	35	1.4	914	35.8
	70–79	1251	518	41	29	2.3	489	39.1
	80+	982	376	38	27	2.8	349	35.5
	All >60	4790	1843	39	91	1.9	1752	36.6
Male	60–69	1945	484	25	85	4.4	399	20.5
	70–79	971	243	25	41	4.2	202	20.8
	80+	508	139	27	30	5.9	109	21.5
	All >60	3424	866	25	156	4.6	710	20.7

**Table 11:** Frequency of abnormal ferritin values in a population-based setting of older adults from the principality of Liechtenstein with the newly established RIs using the direct method for older adults in this study’s ECLIA assay (women: 27–293 ng/mL, men: 23–480 ng/mL).

Sex	Age (y)	N	Abnormal N	Abnormal %	Low Ferritin N	Low Ferritin %	High Ferritin N	High Ferritin %
Female	60–69	2557	333	13	108	4.2	225	8.8
	70–79	1251	247	20	81	6.5	166	13.3
	80+	982	206	21	96	9.8	110	11.2
	All >60	4790	786	16	285	6.0	501	10.5
Male	60–69	1945	339	17	62	3.2	277	14.2
	70–79	971	168	17	27	2.8	141	14.5
	80+	508	107	21	22	4.3	85	16.7
	All >60	3424	614	18	111	3.2	503	14.7

## Data Availability

The raw data supporting the conclusions of this article will be made available by the authors on request.
